# Heat Removal from Bipolar Transistor by Loop Heat Pipe with Nickel and Copper Porous Structures

**DOI:** 10.1155/2014/724740

**Published:** 2014-05-18

**Authors:** Patrik Nemec, Martin Smitka, Milan Malcho

**Affiliations:** Department of Power Engineering, Faculty of Mechanical Engineering, University of Žilina, Univerzitna 1, 01026 Žilina, Slovakia

## Abstract

Loop heat pipes (LHPs) are used in many branches of industry, mainly for cooling of electrical elements and systems. The loop heat pipe is a vapour-liquid phase-change device that transfers heat from evaporator to condenser. One of the most important parts of the LHP is the porous wick structure. The wick structure provides capillary force to circulate the working fluid. To achieve good thermal performance of LHP, capillary wicks with high permeability and porosity and fine pore radius are expected. The aim of this work was to develop porous structures from copper and nickel powder with different grain sizes. For experiment copper powder with grain size of 50 and 100 **μ**m and nickel powder with grain size of 10 and 25 **μ**m were used. Analysis of these porous structures and LHP design are described in the paper. And the measurements' influences of porous structures in LHP on heat removal from the insulated gate bipolar transistor (IGBT) have been made.

## 1. Introduction

**Figure 1:**
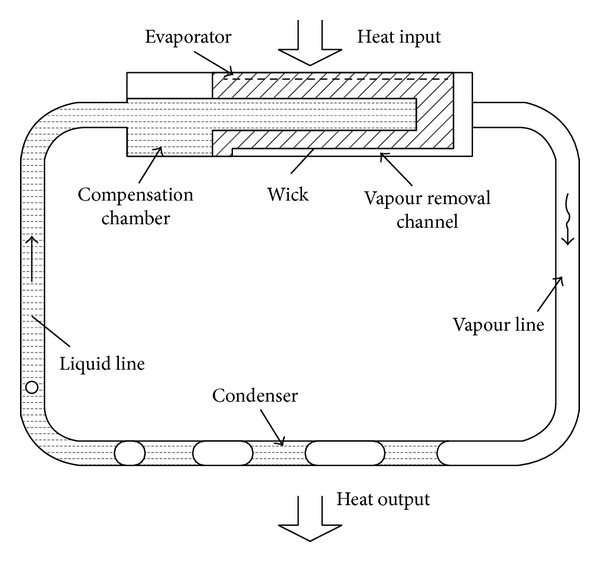
Schematic diagram of LHP [3].

The trend development of electronic components is miniaturization of the dimension. It leads to an increase of waste heat. This heat often leads to lower performance and failure of electronic components in case of insufficient cooling. In order to maintain appropriate working conditions, waste heat must be removed. One possibility to remove waste heat is to use loop heat pipe (LHP). LHPs are two-phase heat transfer devices that utilize the evaporation and condensation of a working fluid to remove heat and the capillary forces developed in fine porous wicks to circulate the fluid. LHP consists of an evaporator with wick, a condenser, a compensation chamber, and liquid and vapor line (Figure 1). Only the evaporator and the compensation contain wicks; the rest of the loop is made of smooth wall tubing. The wick in the evaporator is made with fine pores for purpose of developing capillary pressure to circulate fluid around the loop, while the wick in the compensation chamber is made with larger pores for purpose of managing fluid ingress and egress. The operating principle of the LHP is as follows. As heat is applied to the evaporator, liquid is vaporized and the menisci formed at the liquid/vapour interface in the evaporator wick develop capillary forces to push the vapour through the vapour line to the condenser. Vapour condenses in the condenser and the capillary forces continue to push liquid back to the evaporator. The waste heat from the heat source provides the driving force for the circulation of the working fluid and no external pumping power is required. The two-phase compensation chamber stores excess liquid and controls the operating temperature of the loop [1, 2].

In order for the loop to continue to function, the wick in the evaporator must develop a capillary pressure to overcome the total pressure drop in the loop. One of the advantages of a capillary loop is that the meniscus in the evaporator wick will automatically adjust its radius of curvature such that the resulting capillary pressure is equal to the total system pressure drop. The total pressure drop in the system is the sum of frictional pressure drops in the evaporator grooves, the vapour line, the condenser, the liquid line, and the evaporator wick, plus any static pressure drop due to gravity:

(1) $$\begin{array}{c} {\Delta P}_{\text{total}}={\Delta P}_{\text{groove}}+{\Delta P}_{\text{vap}}+{\Delta P}_{\text{con}}+{\Delta P}_{\text{liq}}+{\Delta P}_{w}+{\Delta P}_{g}. \end{array}$$

The capillary pressure rise that the wick can develop is given by

(2) $$\begin{array}{c} {\Delta P}_{\text{cap}}=\frac{2\sigma \cdot \cos\theta }{R}, \end{array}$$

where *σ* is the surface tension of the working fluid, *R* is the radius of curvature of the meniscus in the wick, and *θ* is the contact angle between the liquid and the wick. As the heat load to the evaporator increases, so will the mass flow rate and the total pressure drop in the system. In response, the radius of curvature of the meniscus decreases so as to provide a higher capillary pressure that matches the total system pressure drop. The radius of curvature will continue to decrease with increasing heat loads until it is equal to the pore radius of the wick, *R*_*p*_. Under this condition, the wick has reached its maximum capillary pumping capability:

(3) $$\begin{array}{c} {\Delta P}_{\text{cap},\max}=\frac{2\sigma \cdot \cos\theta }{R_{p}}. \end{array}$$

Further increase of the heat load will lead to vapour penetration through the wick and system depressed. Thus, under normal operation, the following condition must be satisfied at all times [4]:

(4) $$\begin{array}{c} {\Delta P}_{\text{total}}\leq {\Delta P}_{\text{cap}}. \end{array}$$

Williams and Harris [5] investigated the in-plane and cross-plane properties of step-graded metal felt wicks for heat pipe applications. Porosity, effective pore radius, and liquid permeability were determined using imbibition, capillary flow porometry, and pressure-flow rate data, respectively. The authors determined that many of the correlations in the literature for pore size and permeability are too general in nature, echoing the conclusions of Bonnefoy et al. [6] in regard to effective thermal conductivity.

Holley and Faghri [7] outlined methods for permeability and effective pore radius measurements based on the rate-of-rise test.

Typically, the rate-of-rise test requires observing the liquid front as it rises in a dry wick partially immersed in a liquid pool. As the precise location of this front can be difficult to detect, the authors devised a method using mass uptake rather than the meniscus front to determine the rate of rise of liquid in the wick. By analyzing the climbing meniscus, the authors developed a series of equations which could be used to numerically reduce the mass uptake data to yield permeability and pore size results.

Several relationships for permeability can be found; the most common is the Blake-Kozeny equation [8, 9], which gives the permeability of a bed of packed spheres as

(5) $$\begin{array}{c} K=\frac{r_{p}^{2}\varepsilon^{3}}{37.5{\left(1-\varepsilon \right)}^{2}}, \end{array}$$

where *K* is permeability, *r*_*p*_ is pore radius, and *ε* is porosity.

Ren and Wu [10] modeled the effect of wick effective thermal conductivity in LHP evaporators; a two-dimensional axisymmetric model was developed yielding results in agreement with the literature in some respects, namely, the position of the liquid front in relation to a heated fin [11, 12].

Zhao and Liao [12] present temperature profiles indicating decreasing heat leak for increasing heat flux in a bed of packed spheres.

Iverson et al. [13] studied heat and mass transport in sintered copper wick structures. Wick samples were mounted vertically with the lower section immersed in a pool of water. A heater mounted to the back face of the wick applied power to the sample and the resulting temperature gradients were measured along with the mass flow rate of working fluid.

The majority of heat load is used in vaporization on the outer surface of wick [14]. The rest of heat input (called “heat leak”) is conducted across the wick and is proportional to the effective thermal conductivity (ETC) of the capillary wicks [15]. Lower thermal conductivity of the porous wick ensures lesser heat conduction to the liquid inside the wick inner surface and maintains the operating temperature and thus the thermal resistance of the whole LHP.

Ku [4] and Furukawa [16] in research works dealt with LHP heat leak models utilize conductance parameter and the temperature deference between the evaporator and compensation chamber. Consider

(6) $$\begin{array}{c} {\dot{Q}}_{e,cc}=G_{e,cc}\left(T_{e}-T_{cc}\right), \end{array}$$

where *Q* is heat leak, *G* is conductance parameter, and *T* is temperature of the evaporator and compensation chamber.

In steady state operation, the heat leak to the compensation chamber must be offset by the liquid returning from the condenser; (7) results, where Δ*T* represents the subcooling of the returning fluid

(7) $$\begin{array}{c} {\dot{Q}}_{e,cc}=\dot{m}c_{p}\Delta T, \end{array}$$

where *m* is mass flow and *c*_*p*_ is specific heat.

Chuang [17] developed a steady state LHP model which breaks the overall heat leak into two separate components: axially from the evaporator to the compensation chamber and radially from the heat source to the evaporator core. These two effects are related in that the formation of vapour bubbles in the evaporator core due to radial leak reduces the overall heat flow path back to the compensation chamber, increasing axial leak [4].

Chuang derived the following expressions for the axial and radial heat leak, respectively:

(8) $$\begin{array}{c} {\dot{Q}}_{\text{leak},a}=k_{\text{eff}}A\left(\frac{T_{e}-T_{cc}}{L}\right)+\left(\text{Nu}k_{f}\pi L\right)\left(\frac{T_{e}-T_{cc}}{2}\right),\\ {\dot{Q}}_{\text{leak},r}=\frac{2\pi k_{\text{eff}}L\varsigma }{{\left(r_{o}/r_{i}\right)}^{\varsigma }-1}\Delta T_{w}, \end{array}$$

where *Q*_leak_ is heat leak power, *k*_eff_ is effective thermal conductivity, *A* is area, *L* is characteristic length, Nu is Nusselt number, *k*_*f*_ is fluid thermal conductivity, and *ς* represents a nondimensional ratio of advection and conduction given by

(9) $$\begin{array}{c} \varsigma =\frac{\dot{m}c_{p}}{2\pi k_{\text{eff}}L}. \end{array}$$

In his analysis and experiment, Chuang assumed this parameter to be zero, that is, pure conduction. For the low power cases studying this assumption was valid and resulted in low error; however, for high power levels or low wick conductivity, this assumption loses validity.

## 2. LHP Wick Structure

To achieve good thermal performance, capillary wicks with high permeability and porosity and fine pore radius are expected. These parameters depend mainly on the manufacturing process. The most frequently used wicks are made of sintered metal, like nickel, copper, titanium, stainless steel, or polymers (polyethylene, polypropylene, and PTFE) [18–20].

Tap powder sintering technique using a graphite matrix is used by Reimbrechta et al. [21] to prepare Ni wicks dedicated for capillary pump applications. Graphite is recommended because it shows a low interaction with nickel at the usual sintering temperatures. Xin et al. [22] used two different methods, the cold-pressing sintering and direct loose sintering for development of Ni and Ni-Cu (90% nickel and 10% copper) wicks for loop heat pipes. The optimal Ni-Cu capillary wick is prepared using direct loose sintering technique, with mean pore radii of 0.54 *μ*m. Huang and Franchi [23] fabricated bimodal wick structure using copper screen mesh and two powder materials (nickel filamentary powder and spherical copper powder). But these wicks may be produced with inherent shortcomings. Samanta et al. [24] developed by metal injection moulding Ni wick structures and perform study of its physical characteristic depending on sintering time (30, 60, and 90 min) and temperature (900, 930, and 950°C). Gernert et al. [25] develop fine pore wick structure for LPH. Wu et al. [26] discuss effect of sintering temperature curve in wick structure manufactured for LHP. According to [14] the main parameters of wick are porosity, pore diameter, and permeability. The optimal porosity of sintered wick is between 30 and 75% regardless of the pore diameter. The sintered material porosity increases when the temperature or the forming pressure decreases. The optimal permeability is between 10^−14^ and 3 · 10^−13^ m^2^. The pore diameters of these various porous materials are between 1 and 20 *μ*m, except for copper, which has larger pore diameters (between 20 and 100 *μ*m).

In [22] the optimal capillary wick was found to be sintered at 650°C for 30 min using direct loose sintering technique, with 90% nickel and 10% copper. The wick reaches the porosity of 70% and a mean pore diameter of 1.8 *μ*m. In [4] biporous nickel wicks were fabricated. Porosity of 77.4% was achieved using cold pressure sintering method, at a temperature of 700°C, with a pore former content of 30% in volume.

## 3. Characterization of Sintered Structures

According to the above-mentioned experiences with sintered structures for LHP we decide to make wick structures from nickel and copper powder. At first we do analysis of several sintered structures depending on grain size, sintering temperature and sintering time on porosity, pore size, and strength. In electric furnace etalons from copper powders with grain sizes of 50 and 100 *μ*m and nickel powders with grain size of 10 and 25 *μ*m were sintered. The copper powders were sintered at temperature of 800 and 950°C for time of 30 and 90 minutes and nickel powders were sintered at temperature of 600°C for time of 30 and 90 minutes.

### 3.1. Porosity Measuring

The porosity of a wick structure describes the fraction of void space in the material, where the void may contain working fluid [27]. For the porosity measuring, the weight method was used. At first, the sample was weighed in dry state. Secondly, the sample was soaked with distilled water (*ρ* = 0.998 g·cm^−3^ at 20°C). The weight of absorbed water was estimated by the difference between both values and then a deduction of the “empty space” (thus the total pore volume) and the porosity. Consider

(10) $$\begin{array}{c} \varepsilon =\frac{M_{\text{soaked}\;\text{sample}}-M_{\text{dry}\;\text{sample}}}{V_{\text{total}}.\rho_{\text{distilled}\;\text{water}}}, \end{array}$$

where *ε* is wick structure porosity, *M* is weight of porous sample, *V* is pore volume of the porous sample, and *ρ* is density.

The results of porosity measuring are shown in Tables 1, 2, 3, and 4.

### 3.2. Microscopic Analysis of Pore Size

**Figure 2:**
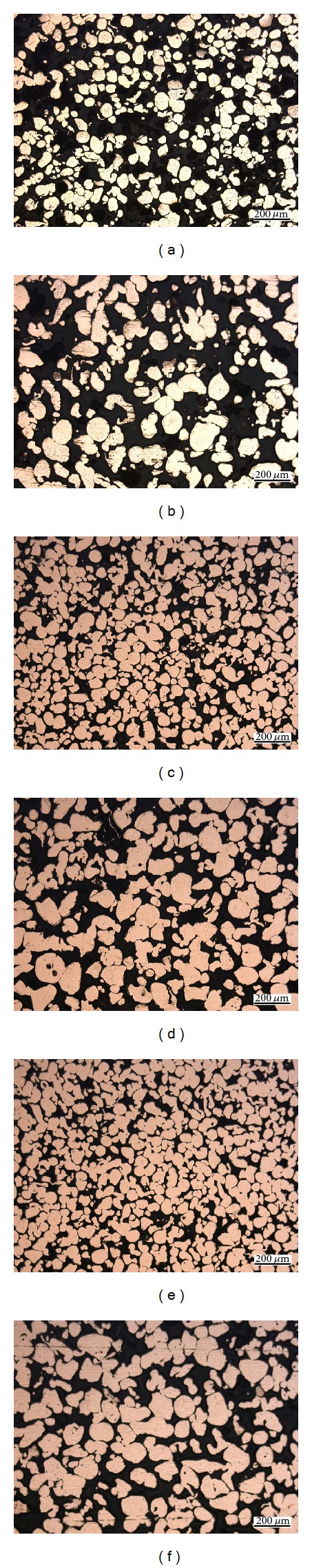
Microscopic pictures of the sintered structures from copper powders. (a) Grain size of 50 *μ*m, sintering temperature of 800°C, and sintering time of 30 min. (b) Grain size of 100 *μ*m, sintering temperature of 800°C, and sintering time of 30 min. (c) Grain size of 50 *μ*m, sintering temperature of 950°C, and sintering time of 30 min. (d) Grain size of 100 *μ*m, sintering temperature of 950°C, and sintering time of 30 min. (e) Grain size of 50 *μ*m, sintering temperature of 950°C, and sintering time of 90 min. (f) Grain size of 100 *μ*m, sintering temperature of 950°C, and sintering time of 90 min.

Investigation of etalons sintered structures by microscopic analysis shown, how influent sintered temperature and time pore size and ratio grain size to pore size of each structure. In Figure 2 are pictures created by 100 times zoom of porous structures sintered from copper powder grain size of 50 and 100 *μ*m. In the first two pictures it is seen that the structures sintered at temperature of 800°C have two times bigger pore than powder grain. Comparison of etalons sintered at temperatures 800 and 950°C shows that the etalons sintered at temperature of 800°C have so much bigger pore size than at temperature of 950°C. It is meaning that pore sizes are so much width to create capillary action in structure. Comparison of etalons sintered at the same temperature and various time intervals observed that the time of sintering at the temperature nearest the melting temperature of sintering material is not decisive. And the last comparison of etalons at the same sintering temperature and time interval observes that the grain size of sintered material has impact on pore size. According to microscopic analysis of sintered structures, which clarifies their shape and profile, we can conclude that the main influencing factor of pore size is grain size, sintering temperature, and not so much sintering time.

**Figure 3:**
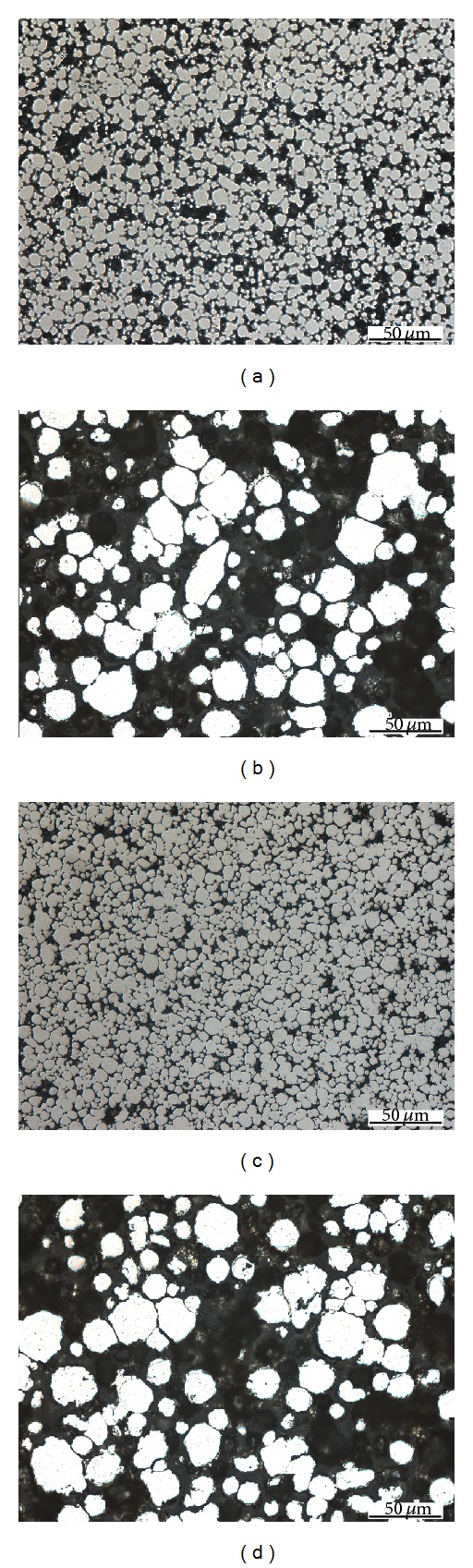
Microscopic pictures of the sintered structures from nickel powders. (a) Grain size of 10 *μ*m, sintering temperature of 600°C, and sintering time of 30 min. (b) Grain size of 25 *μ*m, sintering temperature of 600°C, and sintering time of 30 min. (c) Grain size of 10 *μ*m, sintering temperature of 600°C, and sintering time of 90 min. (d) Grain size of 25 *μ*m, sintering temperature of 600°C, and sintering time of 90 min.

In Figure 3 there are pictures created by 500 times zoom of porous structures sintered from nickel powder grain size of 10 and 25 *μ*m. Comparison of etalons sintered from nickel powder leads to the same conclusion findings as etalons sintered from copper powder. To the formation of pore size sintered structure does not affect sintering time but grain size.

## 4. Design of LHP

**Figure 4:**
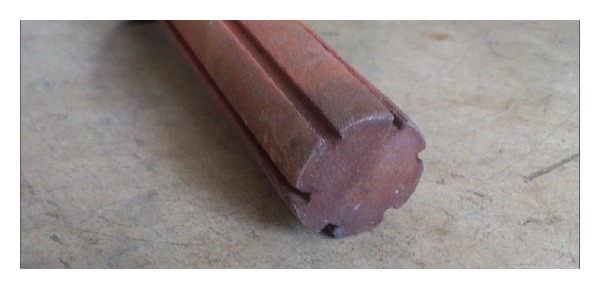
Copper powder sintered wick structure.

**Figure 5:**
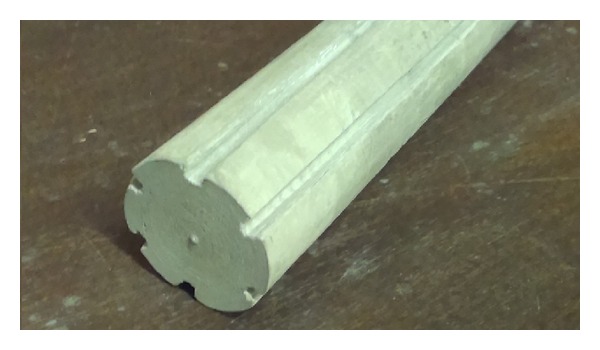
Nickel powder sintered wick structure.

From results of porosity measurement and microscopic analysis for wick structure of LHP two copper etalons and two nickel etalons were chosen. The first structure was made of copper grain size of 50 *μ*m and sintered at temperature 950°C for 30 minutes (Figure 4). The second structure was made of copper grain size of 100 *μ*m and sintered at temperature of 950°C for 30 minutes. The third structure was made of nickel grain size of 10 *μ*m and sintered at temperature of 600°C for 90 minutes (Figure 5). The fourth structure was made of nickel grain size of 25 *μ*m and sintered at temperature of 600°C for 90 minutes. The wick structures were sintered in sand form (mold) manufactured according to model of required shape in muffle furnace.

**Figure 6:**
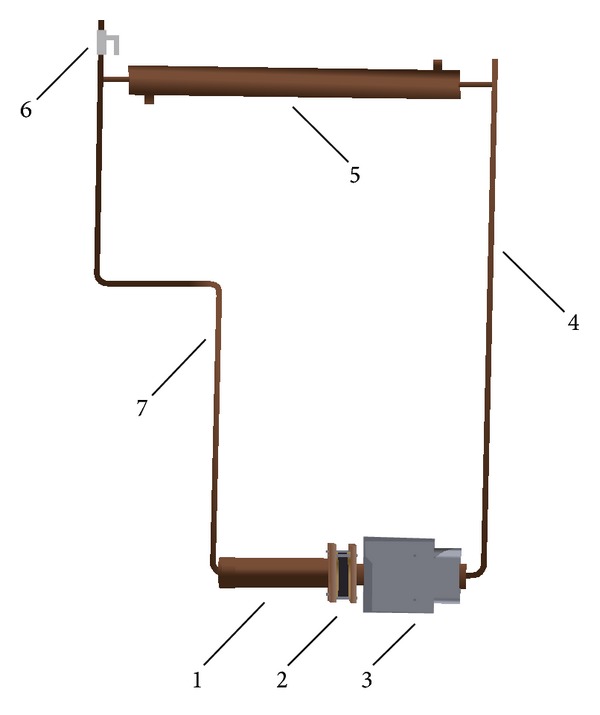
Model of design LHP: 1: compensation chamber, 2: rubber seal, 3: evaporator, 4: vapor line, 5: condenser, 6: filling valve, and 7: liquid line.

All parts of LHP (evaporator, compensation chamber, vapor, and liquid line) were made from copper pipes. As a working fluid distilled water was used. In the evaporator sintered wick structure from copper powder was inserted. To avoid heat loss (it is also called heat leak) into the compensation chamber a brass flange with rubber seal was inserted between the evaporator and the compensation chamber. In Figure 6 there is the model of design LHP and the main parameters of LHP design are in Table 5.

## 5. Measurement and Results

**Figure 7:**
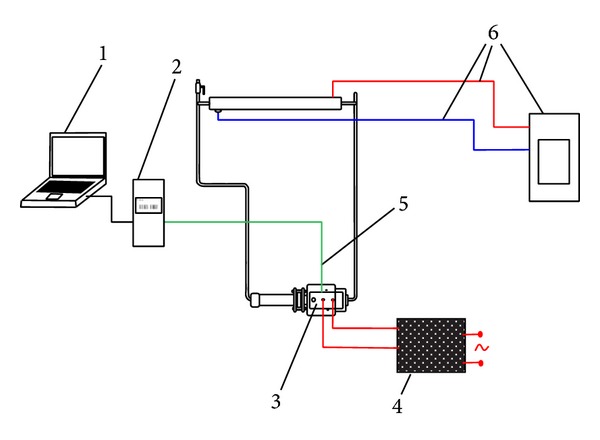
Schematic diagram of measuring device: 1: PC, 2: logger, 3: IGBT, 4: power supply voltage and current, 5: thermocouple, and 6: thermostat.

The LHP with sintered wick structure was proposed to test cooling of IGBT. On the evaporator of LHP the aluminum block with fixed insulated gate bipolar transistor (IGBT) was mounted. For better heat transport thermal conductive paste was applied on the connection between IGBT and aluminum block and between aluminum block and the evaporator [28]. The condenser of LHP was made as tube heat exchanger [29]. The cooling circle of heat exchanger was regulated by the thermostat at constant temperature of 20°C [30, 31]. In Figure 7 there is schema of the measuring unit.

**Figure 8:**
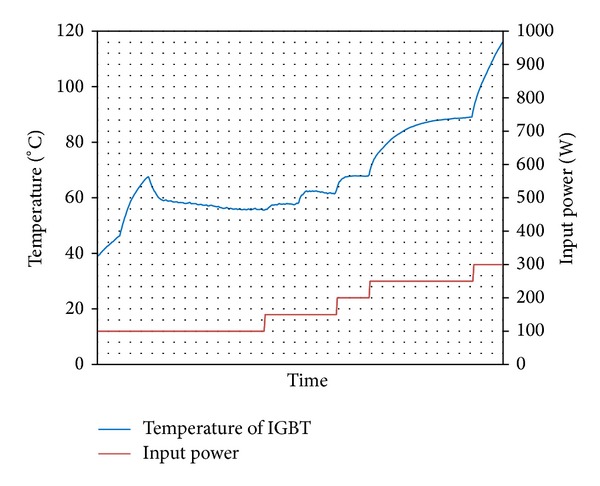
Dependence of temperature on input power of IGBT cooled by LHP with first variant of wick structure.

**Figure 9:**
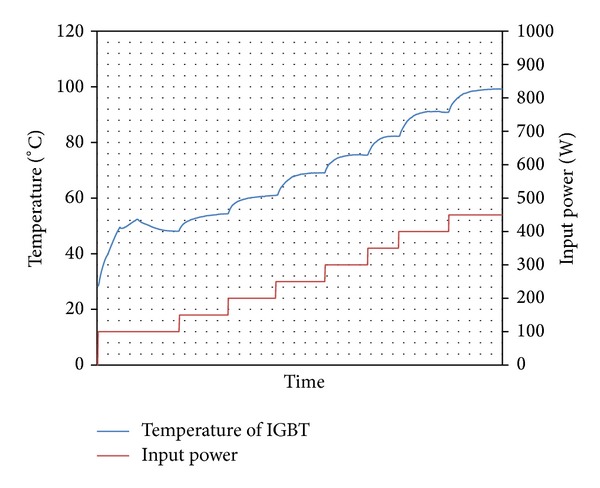
Dependence of temperature on input power of IGBT cooled by LHP with second variant of wick structure.

**Figure 10:**
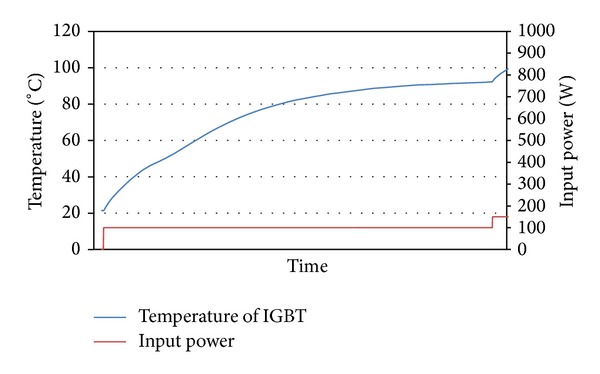
Dependence of temperature on input power of IGBT cooled by LHP with third variant of wick structure.

**Figure 11:**
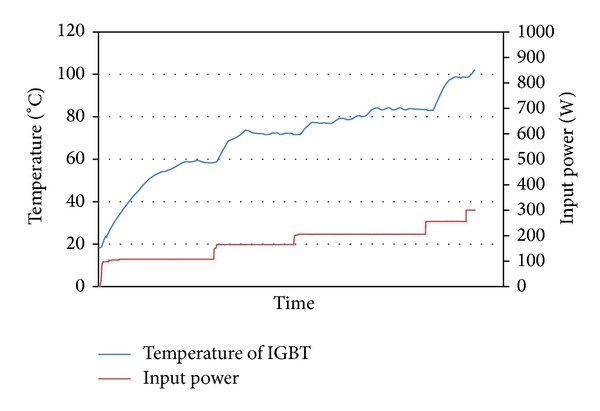
Dependence of temperature on input power of IGBT cooled by LHP with fourth variant of wick structure.

The temperature of the IGBT was measured by thermocouple inserted under IGBT. The maximum permissible temperature of IGBT is 100°C. Transistor was connected to DC power of source and it was gradually loaded by DC. Like this was performed measurement impact of four kinds wick structures in LHP to heat remove from IGBT. The results from measurement of IGBT cooling by LHP with copper wick structures are shown in Figures 8 and 9, and results from measurement of IGBT cooling by LHP with nickel wick structures are shown in Figures 10 and 11. In Figures 8 and 9 it is seen that on start-up of LHP at input power of 100 W the temperature of evaporator increases and only after time, when the LHP starts to operate, the temperature of evaporator decreases and is stabilized. After first stabilization of the temperature input power gradually increased for 50 W. In Figures 10 and 11 it is seen that decrease of temperature did not occur at start-up of LHP with nickel wick structures.

Comparing results of dependence of temperature on input power of IGBT cooled by LHP with various variants of sintered wick structure, the LHP with nickel wick structure did not show better properties of heat removal than LHP with copper wick structure. Comparing dependencies of temperature course on input power of IGBT cooled by LHP with first and second wick structure it is seen that at load of up to 200 W has both LHP almost the same results. At higher input power than 200 W loaded into IGBT it is seen that the LHP with first structure did not remove heat from IGBT and the temperature of IGBT exceeded 100°C. The LHP with second wick structure is able to cool the IGBT under temperature of 100°C until the IGBT input power of 450 W. Comparing dependencies of temperature course on input power of IGBT cooled by LHP with third and fourth wick structure it is seen that evaporator temperature of LHP with third structure at input power of 100 W gradually increases with time and is stabilized at temperature 92°C. The LHP with fourth wick structure is able to cool the IGBT under temperature of 100°C until the IGBT input power of 250 W.

## 6. Discussion

This experiment was performed in framing scientific research of porous structures suitable for LHP and finding ability of heat removal produced by IGBT. We lead off previous research works about LHP in which materials specification suitable for porous structure was preferred. We choose copper and nickel powder with two various granularities. At first etalons were manufactured from each material sintered at various temperatures and times. It was observed that temperature is the main influencing factor on wick structure porosity and pore size is depending on powder grain size. After them for each material one wick structure was manufactured with best characteristics of porosity and pore size and used in LHP for IGBT cooling. The knowledge gained from the IGBT cooling by LHP has given us the information necessary to know how much heat flux is LHP able to remove from heat source. This piece of information will be in the future useful in the design of cooling devices working with the LHP. In the future we would like to focus deeper on analysis of the physical characteristics (e.g., thermal conduction and capillary pressure) of manufactured wick structures and on research of construction design LHP able to remove heat by natural convection to the surroundings.

## 7. Conclusion

According to microscopic analysis of sintered structures, which clarifies their shape and profile, we can conclude that the main influencing factors of pore size are grain size, sintering temperature, and not so much sintering time. The measurement comparison of dependency IGBT temperature from input power cooled by LHP with copper or nickel wick structure can conclude, however, that in both cases the structures had the same porosity and better effect on heat removal from IGBT that had porous structure with bigger pore size. Generally the smallest pore size could cause the low capillary pressure in sintered wick structures against total pressure in whole LHP system.

## Figures and Tables

**Table 1:** Porosity of sintered structures from copper powder with grain size of 50 *μ*m.

Grain size (*μ*m)	50	50	50	50

Sintering temperature (°C)	800	800	950	950

Sintering time (min)	30	90	30	90

Porosity (%)	55	54	52	50

**Table 2:** Porosity of sintered structures from copper powder with grain size of 100 *μ*m.

Grain size (*μ*m)	100	100	100	100

Sintering temperature (°C)	800	800	950	950

Sintering time (min)	30	90	30	90

Porosity (%)	58	56	55	52

**Table 3:** Porosity of sintered structures from nickel powder with grain size of 10 *μ*m.

Grain size (*μ*m)	10	10

Sintering temperature (°C)	600	600

Sintering time (min)	30	90

Porosity (%)	69	67

**Table 4:** Porosity of sintered structures from nickel powder with grain size of 25 *μ*m.

Grain size (*μ*m)	25	25

Sintering temperature (°C)	600	600

Sintering time (min)	30	90

Porosity (%)	72	70

**Table 5:** Main design parameters of the LHP.

Evaporator	
Total length (mm)	130
Active length (mm)	86
Outer/inner diameter (mm)	28/26
Material	Copper
Saddle	
Size (length/height/width)	118/89/40
Material	Alumina
Sintered copper powder	
Number of vapor grooves	6
Porosity (%)	52–55
Outer/inner diameter (mm)	26/8
Sintered nickel powder	
Number of vapor grooves	6
Porosity (%)	67–70
Outer/inner diameter (mm)	26/8
Compensation chamber	
Outer/inner diameter (mm)	35/33
Length (mm)	110
Charge mass	
Distilled water	60%
Vapor line	
Length (mm)	670
Outer/inner diameter (mm)	6/4
Liquid line	
Length (mm)	820
Outer/inner diameter (mm)	6/4
Condenser	
Length (mm)	420
Outer/inner diameter (mm)	6/4
